# Experimental evidence that rill-bed morphology is governed by emergent nonlinear spatial dynamics

**DOI:** 10.1038/s41598-022-26114-0

**Published:** 2022-12-13

**Authors:** Savannah Morgan, Ray Huffaker, Rafael Giménez, Miguel A. Campo-Bescos, Rafael Muñoz-Carpena, Gerard Govers

**Affiliations:** 1 Department of Agricultural and Biological Engineering, University of Florida, Gainesville, FL 32611 USA; 2 IS-FOOD Institute, Public University of Navarre, 31006 Pamplona, Navarre Spain; 3 Department of Earth and Environmental Sciences, KUleuven, Leuven, Belgium

**Keywords:** Hydrology, Applied mathematics

## Abstract

Past experimental work found that rill erosion occurs mainly during rill formation in response to feedback between rill-flow hydraulics and rill-bed roughness, and that this feedback mechanism shapes rill beds into a succession of step-pool units that self-regulates sediment transport capacity of established rills. The search for clear regularities in the spatial distribution of step-pool units has been stymied by experimental rill-bed profiles exhibiting irregular fluctuating patterns of qualitative behavior. We hypothesized that the succession of step-pool units is governed by nonlinear-deterministic dynamics, which would explain observed irregular fluctuations. We tested this hypothesis with nonlinear time series analysis to reverse-engineer (reconstruct) state-space dynamics from fifteen experimental rill-bed profiles analyzed in previous work. Our results support this hypothesis for rill-bed profiles generated both in a controlled lab (flume) setting and in an in-situ hillside setting. The results provide experimental evidence that rill morphology is shaped endogenously by internal nonlinear hydrologic and soil processes rather than stochastically forced; and set a benchmark guiding specification and testing of new theoretical framings of rill-bed roughness in soil-erosion modeling. Finally, we applied echo state neural network machine learning to simulate reconstructed rill-bed dynamics so that morphological development could be forecasted out-of-sample.

## Introduction

**Figure 1:**
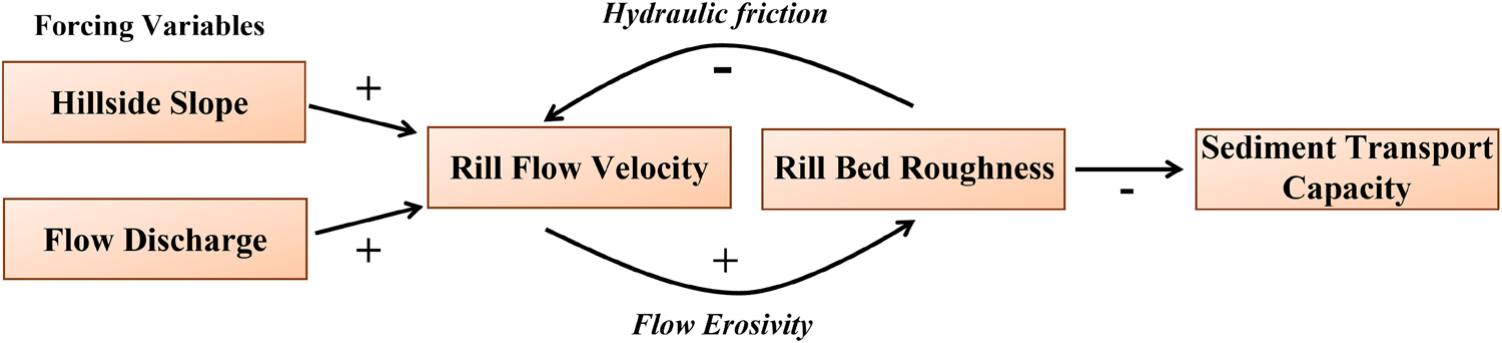
Feedback between rill-flow hydraulics and rill-bed roughness regulate sediment transport capacity. Rill flow velocity increases with hillside slope and flow discharge; rill bed roughness increases due to greater flow erosivity; and rill flow velocity decreases due to greater hydrologic friction, thereby counteracting the initial increasing impact of hillside slope. This feedback mechanism results in reduced erosivity and sediment transport capacity of an established rill.

Overland (sheet) flow of water can rapidly channel into concentrated paths (rills) in response to topography, flow discharge (e.g., precipitation), soil properties, and surface cover^1^. Rill flow causes rapid surface incision due to focused hydraulic power and erosive energy; and consequently, exceeds the sediment transport capacity of sheet flow^1^, accounting for an estimated 80% of sediment eroded from bare hillslopes^2^. Rill erosion is common on steep hillsides where vegetative cover has been compromised by human activities including forestry^1^ and periodic cultivation^3^. Experimental work shows that rill erosion occurs mainly during rill formation^3^ due to feedback between rill-flow hydraulics and rill-bed roughness that regulates the sediment transport capacity of established rills^3–6^. As illustrated in Fig. 1, hillside slope and flow discharge increase rill flow velocity. Rill bed roughness increases with greater flow erosivity when flow and soil conditions combine so that bed geometry freely adjusts to flow hydraulics. The loop is completed as increased rill bed roughness creates hydraulic friction decreasing rill flow velocity, leading to reduced erosivity and sediment transport capacity of an established rill. This counteracts the initial increasing impact of hillside slope on rill flow velocity. Discovery of this feedback mechanism has cast doubt on the implicit assumption of the Universal Soil Loss Equation that sediment transport capacity in rills is well approximated by equations such as Manning’s which hold rill-bed roughness constant^6,7^.

This feedback mechanism typically reshapes the rill-bed into a succession of shallow reaches (steps) and small depressions (pools) characterized by unidirectional and rapidly accelerating flow over steps, and multidirectional and highly turbulent flow that scours pools. The spatial arrangement of step-pool units is critical to rill hydraulics and ultimately rill erosion^1–3,8^. Since conceptual modeling of step-pool units in streams demonstrates that step-pool units evolve towards a maximum flow resistance condition when they are regularly spaced^9^, past work has searched for regularities in the spatial arrangement of step-pool units in rills. No clear regularities have been detected—a result attributed mainly to factors causing local variations in flow and bed resistance that prevent regular step-pool development including rock outcroppings, vegetation, or sudden bends in the rill bed^6,8^.

Experimental rill-bed profiles—spatial series recording longitudinal elevation profiles along a rill bed—exhibit irregular fluctuating patterns of qualitative behavior that conceal underlying spatial dynamics^6^. The conventional perspective is that irregular fluctuations are generated by linear-stochastic dynamics in which steady oscillations randomly shift in response to exogenous shocks^10^. However, recent developments in nonlinear-deterministic dynamics raise an alternative perspective: Irregular fluctuations emerge endogenously in complex systems from nonlinear interactions of system covariates. Complex systems have the capacity for self-organization, from which emerges ordered collective dynamic behavior not exhibited by individual components on their own. Nonlinear dynamics is one of several approaches used to study emergent dynamics since they potentially evolve along a nonlinear attractor—a geometric object bounded within a low-dimensional subset of state space^11^. We emphasize low-dimensionality as a valuable dimension-reducing property: If attractor dynamics of an *n*-dimensional nonlinear dynamic system are bounded within *m* <  < *n* dimensions, the problem of modeling long-term dynamics shrinks by the *n* *− m* inactive degrees of freedom^11^. Long-term system dynamics can be captured with relatively few degrees of freedom regardless of overall system dimensionality without sacrificing essential information. Nonlinear attractors may exhibit irregular and aperiodic dynamic behavior, and thus remain undetected by searches limited to regular periodic behavior. Larsen et al*.* (2014) cite several examples of complex real-world environmental systems with emergent nonlinear-deterministic dynamics^12^.

Rill development is a classic example of a complex system driven by an intricate network of interacting nonlinear climatic, hydrologic, and soil processes; consequently, we hypothesize that rill-bed morphology is driven by emergent nonlinear-deterministic dynamics. To test this hypothesis, we propose an empirical framework centered on a novel application of nonlinear time series analysis^13,14^. Nonlinear time series analysis is designed to reverse-engineer (reconstruct) state-space dynamics from sequential data, and thus distinguish between linear-stochastic dynamics or emergent nonlinear-deterministic dynamics as the most likely source of observed irregular spatial fluctuation in rill-bed profiles. Distinguishing underlying dynamics correctly is essential for statistically reliable soil erosion modeling. Indiscriminately applying linear methods to model nonlinear data creates specification bias: A distorted version of nonlinear variation in the data passes through a linear model to structured residuals, which renders estimation of model coefficients statistically unreliable. The results will set a benchmark guiding specification and testing of new theoretical framings of rill-bed roughness in soil-erosion modeling by providing experimental evidence for whether rill morphology is shaped endogenously by internal nonlinear hydrologic and soil processes or stochastically forced. Our approach deviates from past work which takes measurements of detected step-pool units to identify ‘regular’ dimensional configurations against which candidate step-pool units can be compared^6,8,15^. Alternatively, we reconstruct the spatial dynamics of rill-bed profiles to test whether irregular appearance of step-pool units in experimental profiles is due to internal nonlinear dynamics—not aberrations distorted by external random shocks. Finally, a novel application of echo state neural network machine learning^16^ allows us to simulate empirically-reconstructed rill-bed profile dynamics so that morphological development can be forecasted out-of-sample. The length of short experimental rills can be expanded with machine learning to increase repetition of dominant spatial cycles as a potential remedy for spatial non-stationarity.

### Data

We analyzed fifteen total rill-bed profiles, five resulting from in-situ experiments, and ten from experiments conducted in a flume. The in-situ experiments—described in detail by Giménez et al., 2019—were conducted on approximately 20 × 5 m rectilinear hillslope sections composed of a silt loam topsoil used for crops in Olite, (Navarre, Spain). Each section had a different slope gradient of 3%, 5%, or 15%. In preparation, the surface of each section was smoothed with a gentle central depression inserted so that eroded channels would be as straight as possible. The soil was moistened to saturation and left to drain to field capacity. Next, flow discharge varying from 160 to 5000 Lh^−1^ (Lh^−1^ = Liter per hour) was applied at the top of the slope until steady-state average flow velocity occurred. We label rill-bed profiles by slope and discharge rate, for example, 15 sl–160 Lh^−1^ denotes a slope gradient of 15° and a flow discharge rate of 160 Lh^−1^. In situ rill lengths varied from 4.2 to 15 m (Table 1). The flume experiments—described in detail by Giménez and Govers (2001, 2004)—were formed from freely-developed rill replicas using two different agricultural topsoils: five loamy sand, and five silt loam. Flumes were set at 8° and 12°. Creating the rill involved using a flume 4.50 m long, 0.4 m wide, and 0.45 m deep with a test section 2.3 m in length. The subsoil was simulated through manual compaction of the soil of choice (silt loam or loamy sand) in the lower 0.2 m of the flume and the fine seedbed conditions were simulated by adding the same soil type sieved at 20 mm to the upper 0.25 m of the test section. After simulating the subsoil and fine seedbed conditions, the surface of the plot was smoothed with a rake resulting in a 0.25–0.3 m wide and 50 mm deep longitudinal central depression with a flat bottom. The plot was then moistened to saturation and left to drain to field capacity. Flow discharge rates ranged from 1000 to 3600 lh, and rill lengths from 1.18 to 1.8 m (Table 1).

**Table 1:** Experimental rill-bed profiles and signal processing with singular spectrum analysis.

Rill profile	Length (m)	Detrended signal strength^a^	Oscillatory components
**In-situ rill profiles**			
5 sl–2500 Lh^−1^	11.23	28%	2.03 m (21%), 1.30 m (7%)
5 sl–5000 Lh^−1^	15.08	52%	4.26 m (24%), 5.66 m (28%)
15 sl–160 Lh^−1^	4.220	35%	0.68 m (18%), 0.65 m (10%), 0.61 m (7%)
15 sl–780 Lh^−1^	13.23	38%	0.80 m (15%), 0.50 m (23%)
15 sl–900 Lh^−1^	11.90	39%	0.80 m (25%), 0.20 m (13%)
**Flume (loamy sand) profiles**			
8 sl–1000 Lh^−1^	1.78	77%	0.25 m (68%), 0.28 m (5%), 0.30 m (4%)
8 sl–2200 Lh^−1^	1.79	88%	0.24 m (73%), 0.18 m (11%), 0.30 m (3%)
8 sl–3600 Lh^−1^	1.80	73%	0.16 m (41%), 0.14 m (16%), 0.17 m (16%)
12 sl–1000 Lh^−1^	1.80	77%	0.18 m (37%), 0.13 m (40%)
12sl–2200Lh^−1^	1.80	80%	0.39 m (30%), 0.34 m (30%), 0.25 m (20%)
**Flume (silt loam) profiles**			
8 sl–1000 Lh^−1^	1.18	85%	0.21 m (65%), 0.11 m (20%)
8 sl–3600 Lh^−1^	1.80	76%	0.40 m (64%), 0.66 m (12%)
12 sl–1000 Lh^−1^	1.20	73%	0.28 m (42%), 0.16 m (15%), 0.08 m (15%)
12 sl–2200 Lh^−1^	1.79	86%	0.70 m (43%), 0.25 m (29%), 0.19 m (15%)
12 sl–3600 Lh^−1^	1.79	92%	0.41 m (60%), 0.18 m (21%), 0.39 m (11%)

^a^The percent of total variation in the detrended rill-bed elevation profile accounted for by the detrended signal.

### Data pre-processing

We investigated whether rill-bed profiles resampled at longer sampling intervals along the rill would continue to express full ranges of dynamic behavior with the payoff being reduced computation cost of processing fewer observations. We used the Fourier power spectrum to ensure that resampling to longer intervals did not average out substantial variation. All in-situ and flume rill-bed profiles were resampled by averaging every 10 observations. We found that in-situ rill-bed profiles 5 sl–2500Lh^−1^ and 15 sl–160 Lh^−1^ could be resampled at an interval of 8 mm. The resampling interval of the other three in-situ rill-bed profiles are as follows: 5 sl–5000 Lh^−1^ at 7 mm, 15 sl–780 Lh^−1^ at 21 mm, and 15 sl–900Lh^−1^ at 57 mm. We found that flume rill-bed profiles could be resampled at an interval of 20 mm. We standardized flume rill-bed profiles (by subtracting the profile mean from each observation and dividing by the profile standard deviation), which improved the performance of signal processing in isolating high-frequency oscillations expected to contain step-pool units. Positive (negative) standardized values represent standard deviations above (below) the mean (the zero value). Finally, we filled in sporadic missing observations in rill-bed profiles with the R(*imputeTS*) package.

### Workflow

**Figure 2:**
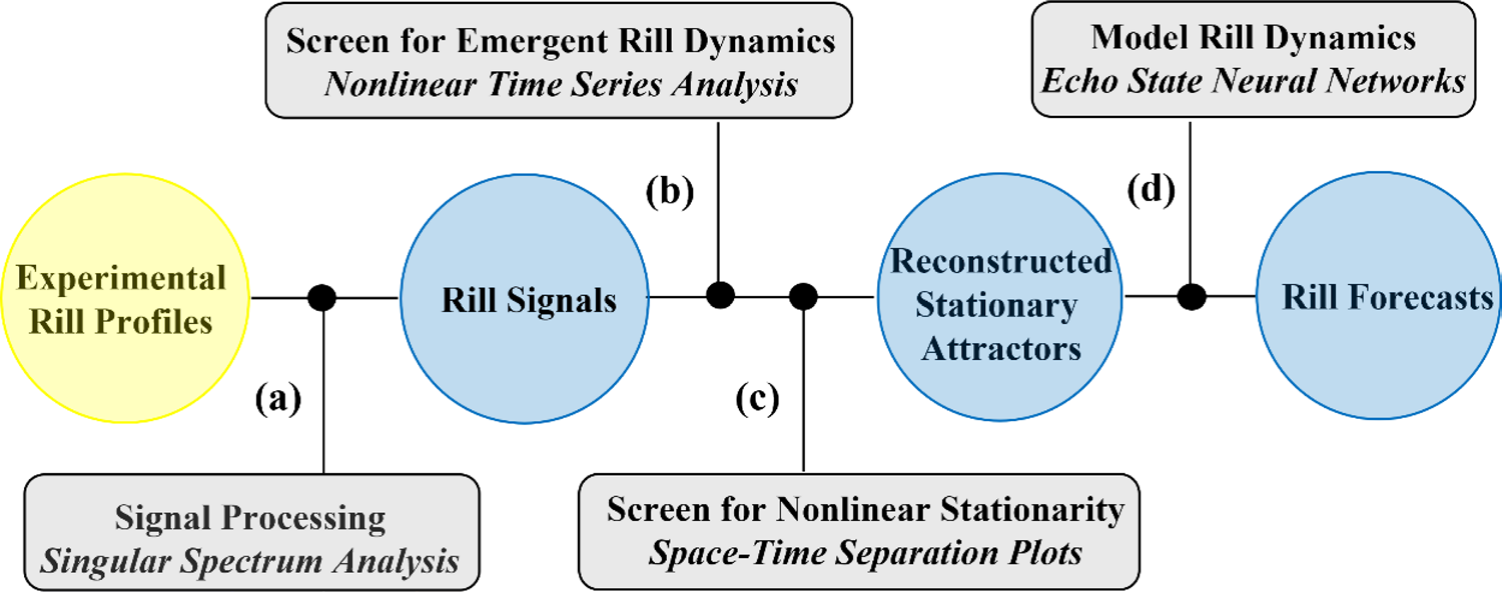
Workflow for reconstructing spatial dynamics from experimental rill-bed profiles. (**a**) We applied singular spectrum analysis to separate signal from noise in rill profiles and remove low-frequency trend components from the signal to isolate higher-frequency cyclical components expected to contain step-pool units and to promote nonlinear stationarity. (**b**) We screened for emergent nonlinear-deterministic dynamics in detrended rill signals with nonlinear time series methods*.* We reverse-engineered (reconstructed) rill-bed morphology dynamics from each detrended rill signal with time-delay embedding, and statistically tested whether apparent nonlinear structure in reconstructed dynamics was likely mimicked by a linear-stochastic process with surrogate data testing. (**c**) We used space–time separation plots to screen for nonlinear stationarity in reconstructed rill-bed dynamics to ensure that rill signals were long enough to adequately sample dominant low-frequency cycles isolated by singular spectrum analysis. (**d**) We applied echo state neural network^16^ machine learning to simulate and forecast reconstructed nonlinear-deterministic dynamics. We used out-of-sample forecasts to increase profile length of rill signals screened to be non-stationarity and re-tested for stationarity.

The workflow followed to reconstruct spatial dynamics from experimental rill-bed profiles and forecast reconstructed dynamics with machine learning is summarized in Fig. 2. First, we applied singular spectrum analysis^17^ to separate signal (structured variation) from noise (unstructured variation) in rill profiles so that we could remove low-frequency trend components from the signal and isolate higher-frequency cyclical components expected to contain step-pool units. Second, we screened for emergent nonlinear-deterministic dynamics in detrended rill signals by reverse-engineering (reconstructing) rill-bed shadow attractors from each detrended rill signal with time-delay embedding^18^, and statistically testing whether apparent nonlinear structure in shadow attractors was likely mimicked by a linear-stochastic process with surrogate data testing^19^. Third, we screened shadow attractors for nonlinear stationarity with space–time separation plots^20^ to ensure that underlying rill signals were long enough to adequately sample dominant low-frequency cycles isolated with singular spectrum analysis. Finally, we simulated and forecasted reconstructed nonlinear-deterministic dynamics with echo state neural network^16^ machine learning. We used out-of-sample forecasts to increase profile length of rill signals screened to be non-stationarity and then re-tested for stationarity.

## Results

### Signal processing

**Figure 3:**
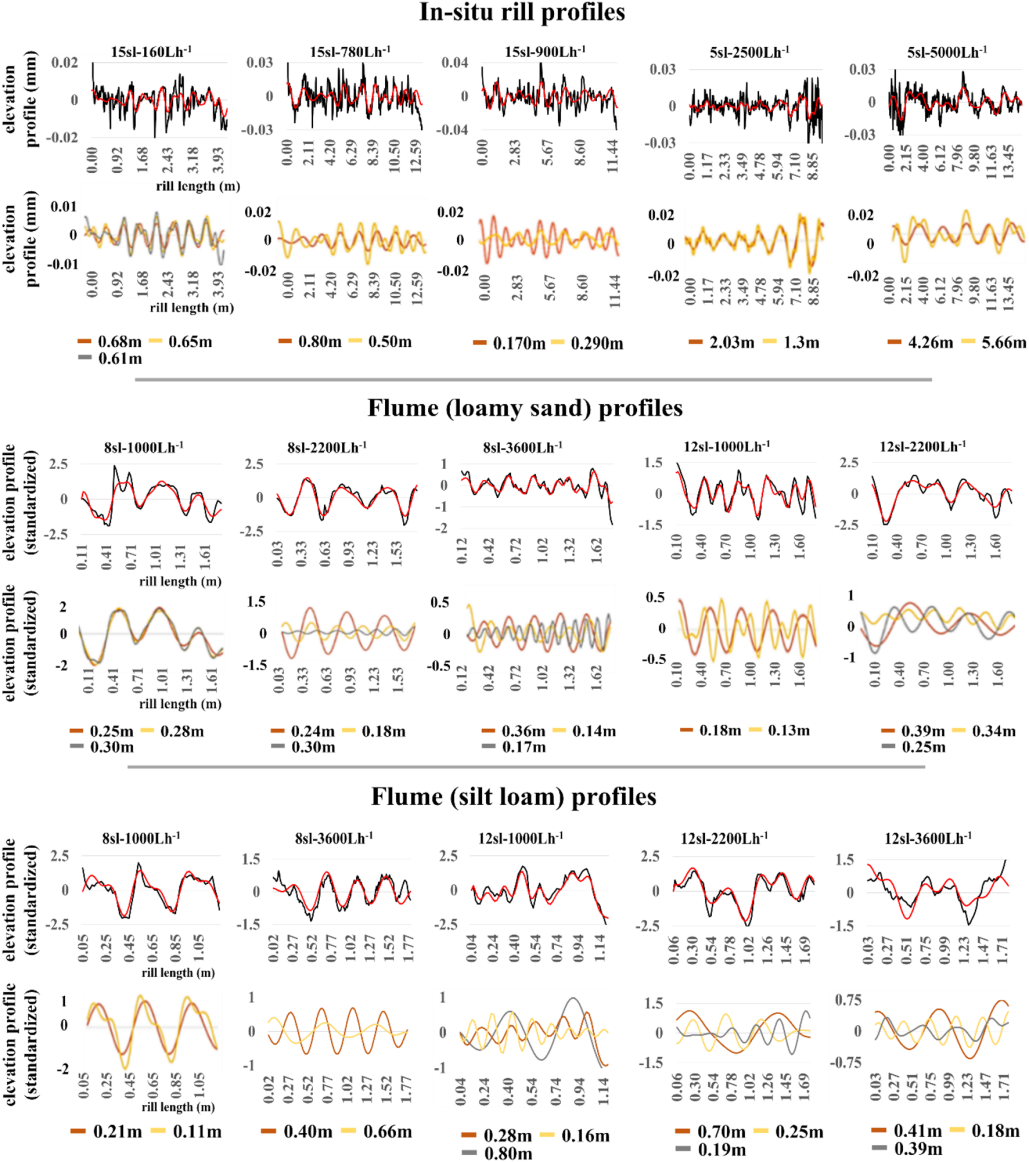
Singular spectrum analysis (SSA) of experimental rill-bed profiles. Upper graphs in each category plot detrended rill-bed profiles (black curve) and the corresponding detrended signal (red curve) against rill length (m); and lower graphs plot corresponding dominant oscillatory components. Removing the hillside-slope trend component from in-situ rill-bed profiles removes steps from step-pool units in the detrended plots; pools remain in the valleys of the detrended profiles. SSA performed better to isolate high-frequency oscillations in flume rill-bed profiles when standardized by subtracting the profile mean from each observation and dividing by the profile standard deviation. Positive (negative) standardized values represent standard deviations above (below) the mean (the zero value). The plots of signals isolated from in-situ and flume rill-bed profiles fluctuate irregularly without exhibiting clear regularities in the spatial distribution of pools.

Table 1 shows that the strength of signals isolated in the detrended in-situ rill-bed profiles ranged from relatively low (28%) to moderate (52%) with four of the five profiles accounting for over a third of total variation in the corresponding detrended profiles. The strength of signals isolated from the ten flume rill-bed profiles were substantially higher (ranging from 73 to 92%) reflecting reduced presence of unstructured noise in a controlled laboratory setting. Signal processing components for in-situ profiles, flume (loamy sand) profiles, and flume (silt loam) profiles are plotted in Fig. 3. For each category, upper graphs plot detrended experimental rill-bed profiles (black curve) and isolated detrended signals (red curve) against rill length (m), and lower graphs plot oscillatory components dominating signals. We observe that filtering out the hillside-slope trend component from in-situ rill-bed profiles removes steps from step-pool units in the detrended plots as seen in Giménez et al., 2019 (compare their figures 1 and 10). Pools remain in the valleys of the detrended profiles. We also observe that the plots of signals isolated from in-situ and flume rill-bed profiles fluctuate irregularly without exhibiting clear regularities in the spatial distribution of pools. The detrended signals are composed of multiple high frequency oscillations (see also Table 1).

### Screen for emergent rill-bed profile dynamics

**Figure 4:**
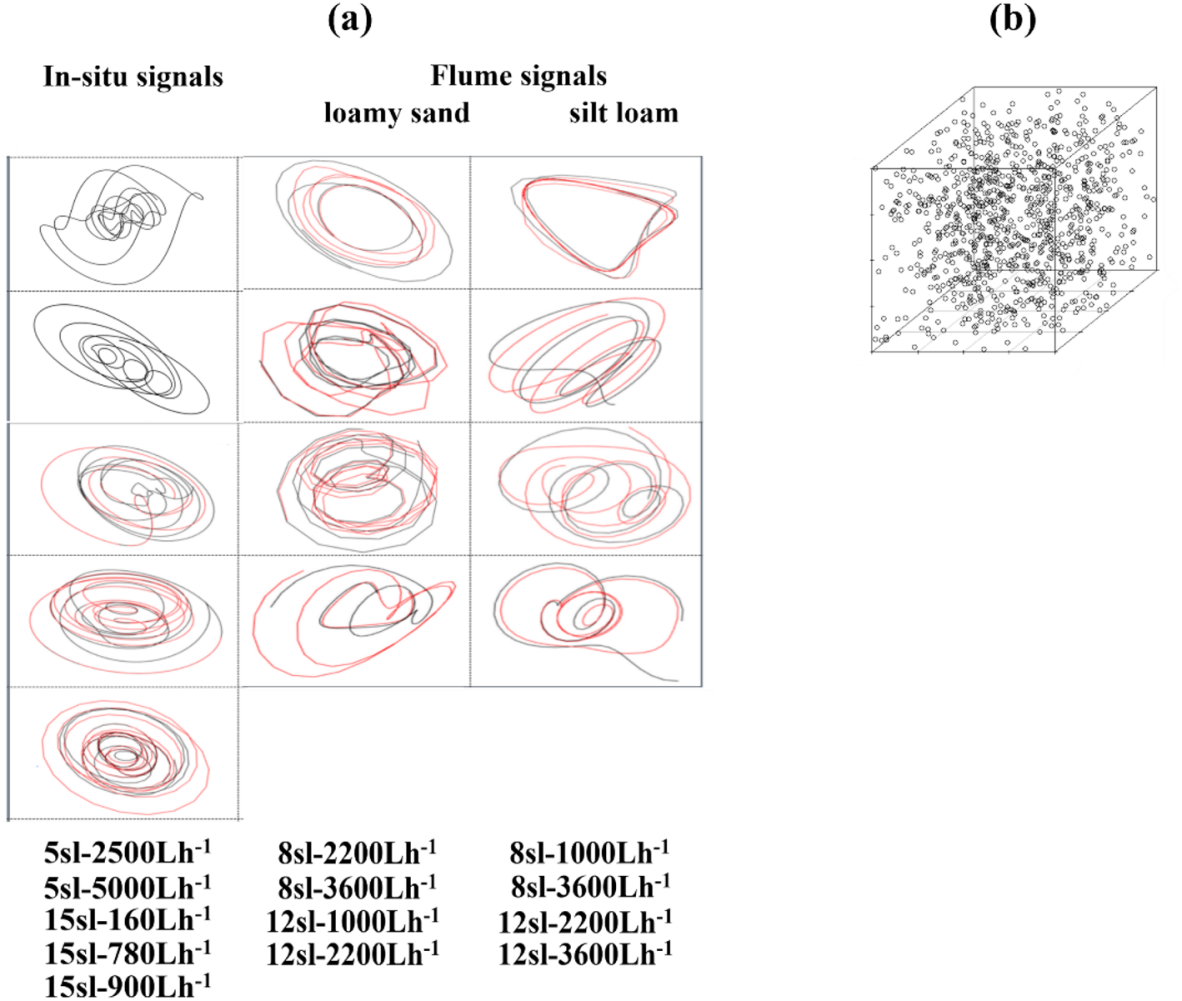
Screening for emergent nonlinear-deterministic dynamics in rill signals. (**a**) We reconstructed low-dimensional shadow attractors from all five in-situ rill signals, and from eight of the ten flume rill signals whose flow discharge levels exceeded 1000 Lh^−1^ (black trajectories). The two failed shadow attractors had too few orbits to adequately sample an underlying attractor, possibly indicating failure of the associated flume rill profiles to develop step-pool units. All reconstructed attractors required at least from two to four embedding dimensions (Table 2), indicating low-dimensional nonlinear dynamics. The plots of shadow attractors have a cyclical appearance composed of aperiodic non-repeating oscillations. In a demonstration of dynamic correspondence, state-space trajectories reconstructed from echo state neural network out-of-sample forecasts (red trajectories) largely rest on shadow attractors reconstructed from in-sample rill signals (black trajectories). Echo state neural network models fit to in-situ rill signals 5 sl–2500 Lh^−1^ and 5 sl–5000 Lh^−1^ were explosive for wide ranges of sampled hyperparameter values, and thus not used for forecasting. (**b**) The visual geometric structure of shadow attractors stands out in contrast to a random scattering of points resulting from reconstruction of a uniform random time series. Surrogate data results soundly reject the null hypothesis that this geometric structure can be attributed to mimicking linear-stochastic dynamics (Table 2).

We reconstructed low-dimensional shadow attractors from all in-situ rill signals from eight of ten flume rill signals. The two failed reconstructions were associated with flume rill signals generated with the lowest discharge level of 1000 Lh^−1^ (8 sl–1000 Lh^−1^ (loamy sand) and 12 sl–1000 Lh^−1^ (silt loam)). The failed shadow attractors had too few orbits to adequately sample an underlying attractor, possibly indicating failure of the associated rill profiles to develop step-pool units. All reconstructed shadow attractors required from two to four embedding dimensions (Table 2), indicating presence of low-dimensional nonlinear dynamics. Three-dimensional projections of shadow attractors (black trajectories plotted in Fig. 4a) have a cyclical appearance composed of aperiodic non-repeating oscillations whose visual geometric structure stands out when compared to a random scattering of points reconstructed from a uniform random time series (Fig. 4b).

**Table 2:** Surrogate data results.

Embedding			Nonlinear prediction test			Permutation entropy test		
Rill profile	m^a^	d^b^	Rill signal *NSE*^c^	PPS surrogates^d^ upper threshold^e^	H0^f^	Rill signal entropy	PPS surrogates lower threshold^g^	H0^h^
**In-situ rill signals**								
5 sl–2500 Lh^−1^	2	20	0.996	− 0.385	Nonlinear	0.399	0.975	Nonlinear
5 sl–5000Lh^−1^	2	20	0.999	− 0.398	Nonlinear	0.310	0.977	Nonlinear
15 sl–160 Lh^−1^	4	14	0.912	− 0.183	Nonlinear	0.739	0.977	Nonlinear
15 sl–780 Lh^−1^	4	16	0.988	− 0.349	Nonlinear	0.97	0.97	Inconclusive
15 sl–900 Lh^−1^	4	5	0.975	− 0.25	Nonlinear	0.607	0.955	Nonlinear
**Flume (loamy sand) rill signals**								
8 sl–2200 Lh^−1^	3	6	0.968	− 0.177	Nonlinear	0.567	0.91	Nonlinear
8 sl–3600 Lh^−1^	4	3	0.788	− 0.064	Nonlinear	0.757	0.91	Nonlinear
12 sl–1000 Lh^−1^	3	3	0.912	− 0.183	Nonlinear	0.739	0.909	Nonlinear
12 sl–2200 Lh^−1^	3	6	0.871	− 0.054	Nonlinear	0.599	0.905	Nonlinear
**Flume (silt loam) rill signals**								
8 sl–1000 Lh^−1^	3	4	0.739	− 0.12	Nonlinear	0.73	0.847	Nonlinear
8 sl–3600 Lh^−1^	3	8	0.979	− 0.123	Nonlinear	0.674	0.909	Nonlinear
12 sl–2200 Lh^−1^	3	4	0.959	− 0.132	Nonlinear	0.703	0.852	Nonlinear
12 sl–3600 Lh^−1^	3	5	0.983	− 0.264	Nonlinear	0.46	0.945	Nonlinear

^a^Embedding dimension; ^b^embedding delay; ^c^Nash Sutcliffe Efficiency (*NSE* = 1 denotes perfect prediction skill); ^d^*PPS* surrogates test the null hypothesis that aperiodic cycling characterizing the empirically-reconstructed attractors is generated by randomly shifting periodic orbits characteristic of noisy linear dynamics. The significance level is set at *α* = 0.05% with 399 surrogates generated; ^e^an upper-tailed test rejects the null hypothesis if the *NSE* computed using the shadow attractor reconstructed from the signal rests above the floor of the upper extreme values computed from surrogate attractors; ^f^Rejection of the null hypothesis leaves the door open to nonlinear-deterministic dynamics; ^g^modified Shannon *H* measure; ^h^a lower-tailed test rejects the null hypothesis if *H* computed using the shadow attractor reconstructed from the signal rests below the floor of the lower extreme values computed from surrogate attractors.

We tested the null hypothesis that apparent geometric regularity in shadow attractors reconstructed from rill signals is due to mimicking linear-stochastic dynamics. We selected nonlinear prediction skill—measured by Nash–Sutcliffe Efficiency (*NSE*)—and permutation entropy—measured by a modification of the Shannon *H* statistic—as discriminating statistics measuring hallmarks of nonlinear-deterministic dynamic behavior. We computed *PPS* surrogate data vectors testing for noisy linear dynamics in cyclical records. We specified an upper-tailed test for nonlinear prediction skill since shadow attractors reconstructed from nonlinear-deterministic data should predict with more skill (larger *NSE*) than attractors reconstructed from 399 *PPS* surrogate data vectors. We formulated a lower-tailed test for permutation entropy since higher *H* values reflect more random behavior. We applied rank-order statistics with significance level α = 0.05 and summarize the results in Table 2. When nonlinear prediction skill is the discriminating statistic, we reject the null hypothesis for all shadow attractors reconstructed from rill signals since the *NSE* achieved by each surpasses the corresponding upper-threshold value exceeded by the top ranked surrogate attractors. When permutation entropy is the discriminating statistic, we reject the null hypothesis for ten of eleven shadow attractors since *H* computed for each falls below the corresponding lower-threshold value bounding from above the bottom ranked surrogate attractors. The permutation entropy test was inconclusive only for the shadow attractor reconstructed from rill signal 15 sl–780 Lh^−1^ since *H* equals the lower-threshold value. Given the strength of these results, we confidently reject the null hypothesis that linear-stochastic dynamics are the most likely source of geometric regularity in shadow attractors reconstructed from rill signals. Nonlinear-deterministic dynamics remain a likelihood.

### Screen for nonlinear stationarity

**Figure 5:**
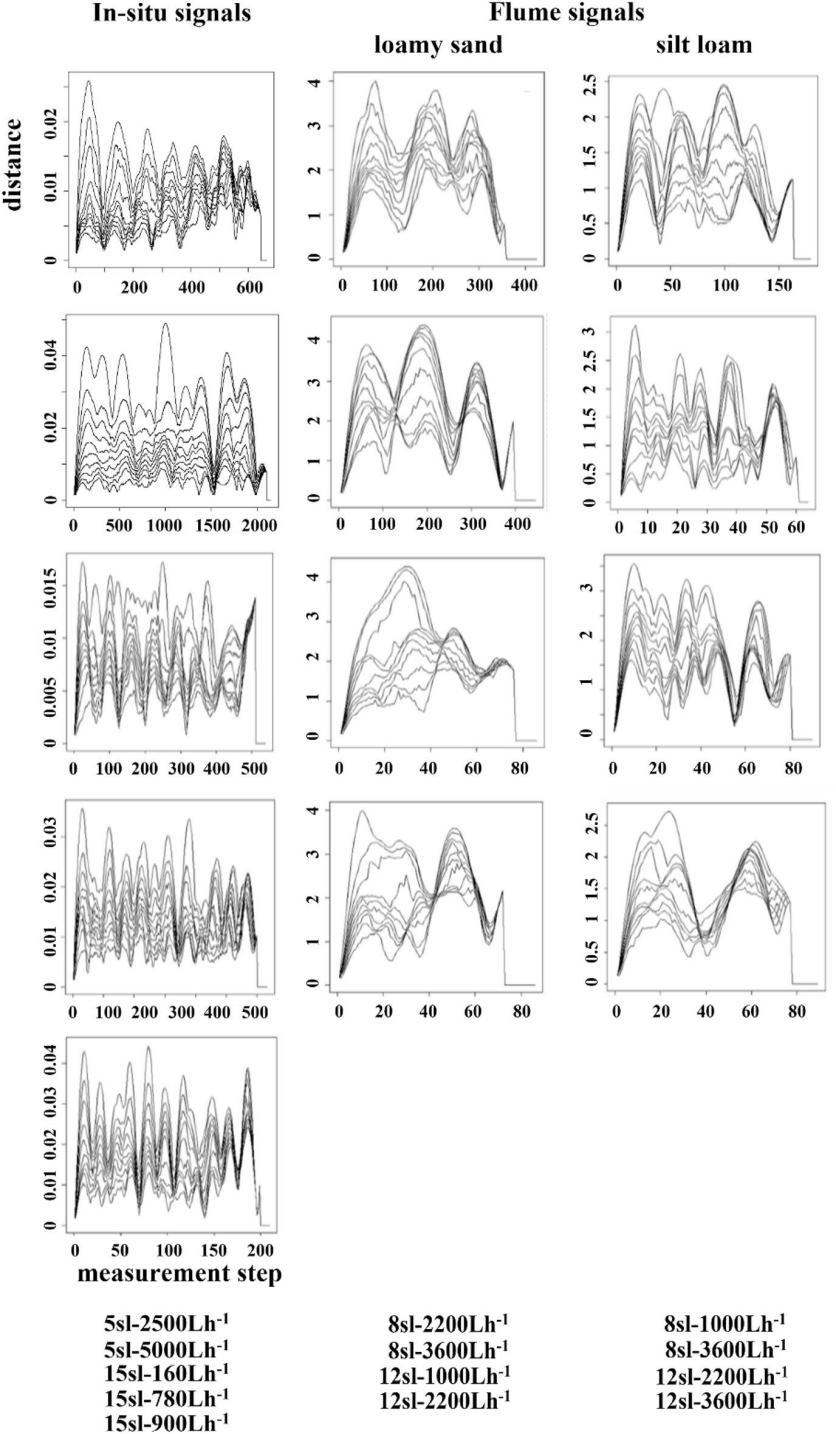
Screening for stationary nonlinear rill-bed dynamics with space–time separation plots. The plots indicate non-stationarity of three flume shadow attractors: 12 sl–1000 Lh^−1^ (loamy sand), 12 sl–2200 Lh^−1^ (loamy sand), and 12 sl–3600 Lh^−1^ (silt loam) since contour cycles require many measurement steps to complete in contrast to contour cycles in the other plots oscillating with much higher frequency. Since these flume rill profiles may be non-stationarity due to relatively short lengths (ranging from 1.18 to 1.80 m in Table 1), we sought to remedy this by increasing profile length with out-of-sample machine-learning forecasts.

Nonlinear **s**tationarity requires that the “duration of the measurement is long compared to the time scales of the systems”^21^. Consequently, an important implication of finding rill signals to be stationarity is that they are long enough to adequately sample the dominant low-frequency cycles isolated by singular spectrum analysis. Space–time separation plots exhibiting contour cycles requiring large numbers of measurement steps for completion indicate that measurement distance between points on an attractor continues to affect their Euclidean distance—a sign of spatial non-stationarity of the signals from which the attractors were reconstructed. Contour cycles in the space–time separation plots for three flume shadow attractors reconstructed from 12 sl to 1000 Lh^−1^ (loamy sand), 12 sl–2200 Lh^−1^ (loamy sand), and 12 sl–3600 Lh^−1^ (silt loam), require large numbers of measurement steps for completion in contrast to contour cycles in the other plots oscillating with much higher frequency (Fig. 5). To address the possibility that these flume rill-profile signals were prone to non-stationarity because of their relatively short lengths (ranging from 1.18 to 1.80 m in Table 1), we increased profile length with out-of-sample machine-learning forecasts and retested for stationarity as reported below.

### Model reconstructed rill-bed dynamics with machine learning

**Figure 6:**
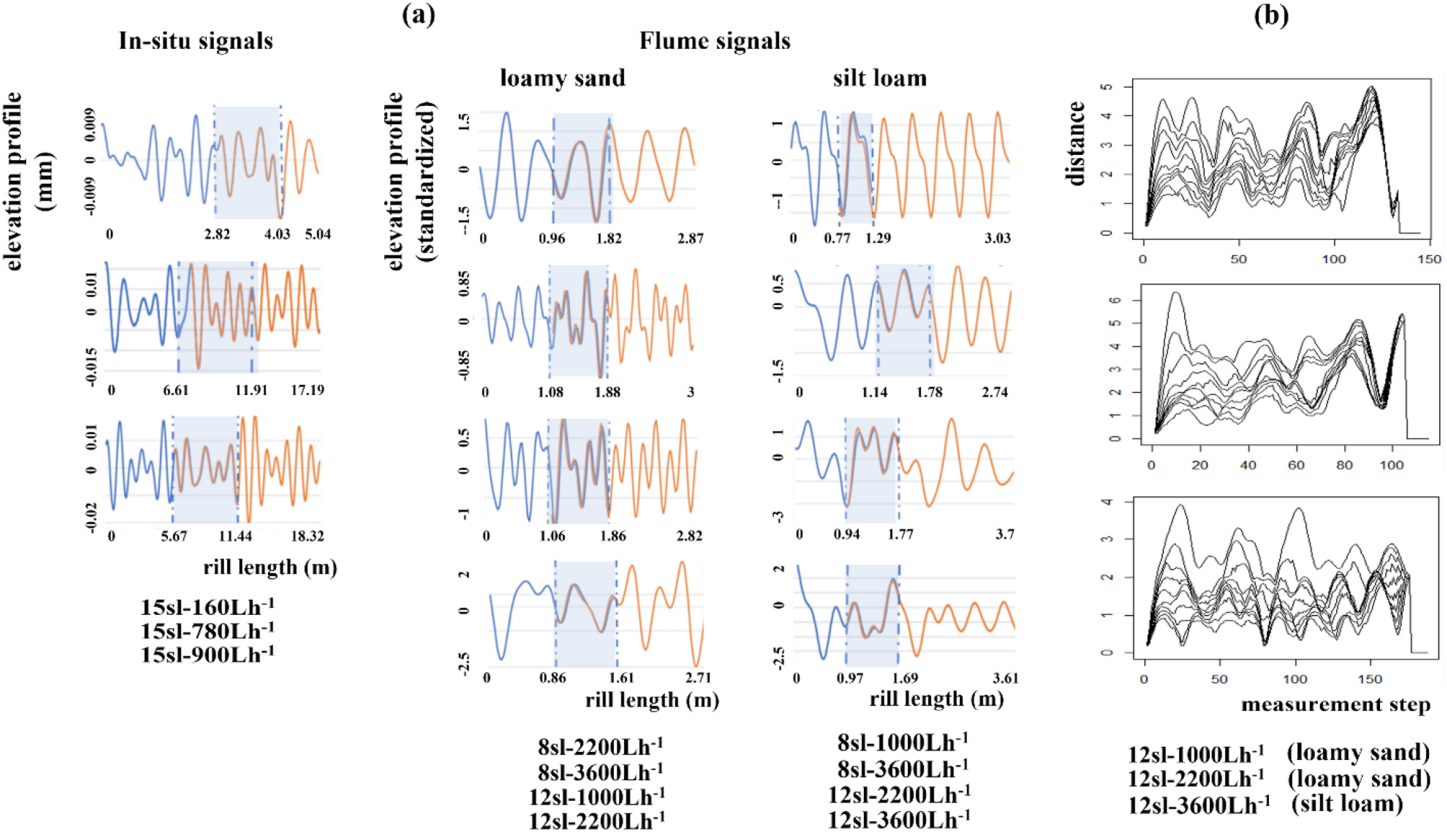
Modeling shadow attractors with Echo State Neural Network (ESNN) machine learning. (**a**) Each performance plot shows the rill signal in the training set (blue curve to left of shaded area), the rill signal in the testing set (blue curve within the shaded area), ESNN in-sample predictions (orange curve within shaded area), and ESNN out-of-sample forecasts (orange curve to right of shaded area). The shaded area represents the testing interval in which ESNN predictions (orange curve) are plotted with the rill signal in the testing set (blue curve). In each plot, ESNN predicts with almost-perfect skill (*NSEs* > 0.95) as evidenced by orange curves overlapping blue curves in the shaded area. Forecasts largely preserved oscillatory behavior observed in corresponding rill profile signals. (**b**) We used ESNN forecasts to extend the lengths of three non-stationary flume rill signals and re-tested for stationarity with space–time separation plots. The plots show that the extended rill signals are now stationary.

Echo state neural networks (ESNN) learned shadow attractors reconstructed from three of five in-situ rill signals (15 sl–160 Lh^−1^, 15 sl–780 Lh^−1^, 15 sl–900 Lh^−1^), and all shadow attractors reconstructed from flume rill signals. ESNN failed to learn reconstructed dynamics from in-situ rill signals 5 sl–2500 Lh^−1^ and 5 sl–5000 Lh^−1^ since runs were explosive for wide ranges of sampled hyperparameter values. In Fig. 6a, we show performance plots for the successful ESNN simulations. The plots focus on rill signals as the first coordinate vector of shadow attractors reconstructed with time-delay embedding. Each plot shows the portion of the rill signal allocated to the training set (blue curve to left of shaded area), the portion remaining in the testing set (blue curve within the shaded area), ESNN in-sample predictions (orange curve within shaded area), and ESNN out-of-sample forecasts (orange curve to right of shaded area). The shaded area represents the testing interval in which ESNN skill in learning rill-signal dynamics is demonstrated by how close ESNN predictions (orange curve) track the rill signal in the testing set (blue curve). In each plot, ESNN predicts with almost-perfect skill (*NSEs* > 0.95) as evidenced by orange curves effectively covering the blue curves. We used the trained ESNN models to forecast each rill signal out-of-sample. Forecasts largely preserved oscillatory behavior observed in corresponding rill profile signals. Moreover, in a demonstration of dynamic correspondence, state-space trajectories reconstructed from ESNN out-of-sample forecasts (red trajectories) largely rest on shadow attractors reconstructed from in-sample rill signals (black trajectories) (Fig. 4).

We used ESNN forecasts to extend the lengths of the three flume rill signals detected to be non-stationarity above: 12 sl–1000 Lh^−1^ (loamy sand), 12 sl–2200 Lh^−1^ (loamy sand), and 12 sl–3600 Lh^−1^ (silt loam). We re-tested for stationarity with space–time separation plots, which revealed that the extended rill signals are now stationary (Fig. 6b). Extended rill lengths became long enough to more adequately sample the dominant low-frequency cycles isolated in signal processing.

## Discussion

Past experimental work found that rill erosion occurs mainly during rill formation in response to feedback between rill-flow hydraulics and rill-bed roughness that creates a succession of step-pool units self-regulating the sediment transport capacity of established rills. Recent work has searched for clear regularities in the succession of step-pool units without success. The intuition that rill morphology might self-organize into systematic spatially dynamic behavior was on the right track, but the search needed to be broadened to enable detection of irregular and aperiodic nonlinear-deterministic spatial dynamics characteristic of complex systems. Our results provide compelling experimental evidence supporting the hypothesis that the succession of step-pool units is governed by emergent nonlinear-deterministic spatial dynamics; and consequently, that rill morphology is shaped endogenously by internal nonlinear hydraulic and soil processes rather than stochastically forced. We successfully reconstructed low-dimensional nonlinear shadow attractors from thirteen of fifteen rill signals investigated, all of which held up against surrogate data testing. We were unable to reconstruct shadow attractors from two flume rill signals generated by the lowest discharge level (1000 Lh^−1^)—likely because these slope and discharge levels precluded development of systematic step-pool units. Low estimated embedding dimensions provide justification for modeling long-term rill-bed dynamics with relatively few degrees of freedom regardless of the immense size and complexity of the real-world system generating experimental rill-bed profiles. This is good news for future efforts to replace constant rill-bed roughness coefficients in conventional soil erosion modeling with parsimonious spatially dynamic rill-bed models. Our results are robust across the rill-bed profiles investigated since emergent nonlinear spatial dynamics were detected in: (1) profiles generated both in a controlled lab (flume) setting and in an in-situ real-world hillside setting; and (2) flume profiles created in both silt-loam and loamy-sand soils. Compatible with Giménez and Govers (2001), this suggests that the spatial arrangement of step-pool units (macroroughness) is not largely conditioned by soil type so long as the soil is susceptible to erosion (erodible material).

Our results contribute more broadly to a growing literature demonstrating that many types of erosional landscapes (e.g., channel bed-steps in ephemeral reaches of boulder and bedrock streams^22^, undulating canyon walls^23^, bedrock waterfalls^24^, and cyclic steps in erodable surfaces^25^ including emphemeral gullies^26^) are shaped by internal (autogenic) dynamics governing feedbacks among topographical, erosional, and sediment-transport processes. Zeng et al. (2021) found that gully erosion is characterized by cyclic steps that are comparable to step-pool units in rill erosion, except that “cyclic steps can form autogenically on homogeneous bed surfaces of uniform bed material.” We offer experiental evidence that step-pool units in rill erosion also form autogenically. We further address a critical research gap identified in a review by Scheingross et al. (2020)^27^. The authors concluded that, although autogenic dynamics are increasingly detected in depositional systems, understanding remains nascent because criteria are lacking to distinguish internal dynamics from external forcing. We conceptualized rill erosion as a complex system in which system variables are internalized by an extensive web of nonlinear interactions. Each variable encodes its internal interactions with covariates, which famous naturalist John Muir intuited in the early nineteenth century when he observed that: “When we try to pick something up by itself, we find it hitched to everything else in the universe”^28^. This precludes the need to synthetically separate internal from external variables. Instead, we isolated structured variation (signal) in experimental rill profiles from unstructured variation (noise) with signal processing, and empirically detected emergent autogenic dynamics in signals with nonlinear time series analysis.

There are important caveats in applying nonlinear time series analysis to reconstruct dynamics of real-world systems from experimental datasets. Records are often noisy and short. Noise must be carefully filtered from records without unintentionally removing aperiodic nonlinear dynamic structure mistaken for noise. We applied singular spectrum analysis to remove noise from irregular appearing rill profiles because this method retains aperiodic oscillations in the isolated signal. Records that are too short to adequately sample the dominant low-frequency cycles isolated by singular spectrum analysis violate nonlinear stationarity requirements. We tested rill-bed profiles for nonlinear stationarity with space–time separation plots. We mitigated detected non-stationarity in in-situ rill-bed profiles by filtering out inadequately sampled low-frequency trend cycles with multi-stage singular spectrum analysis, and in flume rill-bed profiles by extending rill lengths with out-of-sample forecasts computed with machine learning. Even with these precautions, we cannot reasonably expect to reconstruct the complex folding and fractal patterns of real-world attractors^29^, but must lower our expectations to reconstruct a sampling or skeleton of an attractor^30^. We will fall short of reconstructing even a skeleton attractor if: (1) a low-dimensional real-world attractor does not exist; or (2) the data sample only transitory dynamics heading toward an attractor.

We emphasize that nonlinear time series analysis does not replace conceptual modeling of soil erosion based on first principles. Rather, the broader impact of our work is to provide a rigorous empirical benchmark guiding specification and testing of new theoretical framings of rill-bed roughness in next-generation modeling. This benchmark includes a geometric picture of rill-bed profile dynamics that conceptual models should reproduce, and an estimate of the minimum model dimensionality required to do so.

## Methods

### Signal processing

Singular spectrum analysis is a data-adaptive signal processing method accommodating highly anharmonic (aperiodic) oscillations in irregular records^14,17^. It is used to separate structured variation (signal) composed of trend and oscillatory components from unstructured variation (noise). The strength of each component is based on its contribution to explaining total variation in the record. We searched for emergent nonlinear dynamics in the structured signal component of measured rill-bed profiles since including unstructured noise obscures detection. Singular spectrum analysis can be run in multiple stages to remove low-frequency components that: (1) violate stationarity conditions requiring that the “duration of the measurement is long compared to the time scales of the systems”^31^; or (2) impede detection of fainter higher-frequency oscillatory behavior. We ran three-stage singular spectrum analysis on the in-situ rill-bed profiles to remove low-frequency trend components. The 1^st^ stage removed the dominant trend due to hillside slope, the 2^nd^ stage removed lingering trend components driven by physical processes beyond hillside slope, and the 3^rd^ stage isolated higher frequency components expected to capture finer structure in rill morphology containing step-pool units.

### Time-delay embedding

We applied time-delay embedding^18^ to reconstruct a shadow attractor from each detrended rill signal. The matrix form of a shadow attractor (embedded data matrix) is composed of a first column containing the observed rill signal and remaining columns containing space-delayed copies of the observed signal which serve as surrogates for omitted system variables. The number of columns in the embedded data matrix is the embedding dimension, and the delay length between columns is the embedding delay. The columns of the embedded data matrix are coordinates axes in state space, and the rows are multidimensional points on a shadow attractor. Takens (1980) formally proved that time-delay embedding provides a 1–1 mapping of system dynamics from the original real-world state-space to the reconstructed shadow state space so long as the latter has sufficient dimensions to contain the original attractor. Since we do not directly observe the dimension of the real-world attractor, we followed convention in estimating the embedding dimension with the false nearest neighbors test^32^*,* and the embedding delay as the delay giving the first minimum of the mutual information function^32^.

### Surrogate data testing

We tested the shadow attractor reconstructed from each rill signal against *surrogate data* to provide a statistical safeguard against mistaking apparent geometric regularity in a shadow attractor for deterministic nonlinear dynamic structure when it is most likely mimicked by linear stochastic dynamics^19,33^. First, we generated surrogate data vectors that destroyed the spatial structure of rill signals while preserving statistical properties compatible with a hypothesized stochastic dynamic structure. We computed PPS surrogates which test for noisy linear dynamics in cyclic records^34^. Second, we reconstructed attractors from each surrogate data vector and compared them to the shadow attractor reconstructed from a rill signal based on two discriminating statistics conventionally measuring hallmarks of nonlinear-deterministic behavior: nonlinear predictive skill^35^ and permutation entropy^36^. In a conventional nonlinear prediction algorithm, the points on a shadow attractor are split into learning and testing sets, the last point in the training set is predicted one-step-ahead by taking a weighted average of the nearest neighboring points in the training set, and skill measured by how close the prediction is to the corresponding point on the attractor in the test set^35^. We specified an upper-tailed hypothesis test for nonlinear predictive skill since nonlinear-deterministic structure should predict better with a nonlinear prediction algorithm than linear-stochastic surrogates measured by a goodness-of-fit measure such as the Nash–Sutcliffe Model Efficiency (*NSE*)^37^, which denotes perfect skill when *NSE* = 1. Permutation entropy modifies the classic Shannon *H* measure of the information in a time series for application to finite noisy data. When *H* = 0, the time series is perfectly predictable from past values. *H* achieves a maximum value when time series observations are independent and identically distributed. We adopted a lower-tailed hypothesis test for permutation entropy since larger values of *H* indicate more random behavior. Third, we applied rank-order statistics to test for significant difference in nonlinear performance^33^. We generated an ensemble of *S* = (*k*/*α*) − 1 surrogates, where *α* is the probability of false rejection and *k* controls the number of surrogates and the sensitivity of the test. We set *α* = 0.05 and *k* = 20 and accepted the null hypothesis of stochastic cycling dynamics if the *NSE* (*H*) taken from the attractor reconstructed from the rill signal did not fall in the upper (lower) *k* corresponding values taken from the ensemble of *S* = 399 surrogate attractors. If we reject the null hypothesis, untested dynamic structures (including nonlinear-deterministic dynamics) remain viable.

### Screen for nonlinear stationarity

We tested for nonlinear stationarity with space–time separation plots^20^, which scatterplot the Euclidean distance (vertical axis) and the number of measurement steps (horizontal axis) between each pair of points on a shadow attractor. Scatterplotted distances are conventionally reformatted as equal-probability contour lines by plotting the percentage of pairs that are less than or equal to a given Euclidean distance and drawing curves through identical percentages. In the simplest plots, these contours saturate, or in more complex plots, cycle. Contour cycles requiring large times for completion indicate that measurement distance between points on an attractor continues to affect their Euclidean distance—an indication of non-stationarity.

### Model reconstructed rill dynamics with machine learning

Echo state neural network^16^ (ESNN) machine learning is a reservoir computing method comprised of a reservoir mapping each point on the attractor sequentially into a high-dimensional space, and a read-out providing reservoir predictions. Reservoir computing is fast-learning with low-training cost since the reservoir is fixed and only the readout is trained. The embedded data matrix of a reconstructed attractor is divided into training and testing sets. In each iteration *t* of the training mode, a row of the training set (i.e., a point on the attractor) is inputted through an input coupler into a reservoir composed of *N* neurons, $x_{i} (t)\,,\,\,\,i = 1,2,...N$. The scalar activation values are updated in each iteration and collected in a neuron activation matrix, *X*. The training set is regressed against *X* to estimate a coefficient matrix, $W_{out}$, used to formulate the read-out component. In each iteration of the testing mode, a row of the testing set is inputted into the reservoir, and the linear read out generates predicted values, $y(t)\, = \,W_{out} x(t)$, where *x*(*t*) is the vector of activations at iteration *t*. The neuron activations are updated at each iteration, and the updated predictions collected in prediction matrix *Y*. In sum, *Y* is the ESNN simulation of the portion of the embedded data matrix allocated to the testing set. Consequently, the first column of *Y* is the prediction of the rill profile signal contained in the first column of the embedded data matrix, and the rows of *Y* are predicted points on the testing portion of the reconstructed attractor. We measure how well each column of *Y* predicts its counterpart in the embedded data matrix with the Nash–Sutcliffe Efficiency Index^37^. Since machine learning performance is highly sensitive to architectural hyperparameters^16^, we run ESNN through a ‘tuning cycle’ that employs high-performance computing (HPC) and global sensitivity analysis^38^ (GSA) to automate a large parallel grid search of architectural hyper-parameters. The tuning cycle first uniformly samples a hyper-parameter grid with option for sparse Morris or denser Sobol GSA sampling methods^38^. Sampled machine learning configurations are parallelized and batch run with *HPC*. Post-processing GSA identifies which hyper-parameters are most influential in driving machine learning performance, and consequently can be effectively adjusted in another tuning cycle. If we select the Morris sampling method, we compute Morris sensitivity measures; if we select the Sobol method, we compute first-order and total-sensitivity variance-decomposition indices^38^. In generation mode, we use skillful parameterizations to forecast points on the attractor out-of-sample by feeding back in predicted values at each iteration.

## Data Availability

The data sets used and/or analyzed during the current study are available from the corresponding author.

## Code availability

These R packages were used: imputeTS (fill in data gaps), RSSA (singular spectrum analysis); spacetime (spacetime separation plots); and tseriesChaos (mutual information function, false nearest neighbors test, time-delay embedding). These packages are downloaded from https://cran.r-project.org/package = *, where * is a package. Wrap-around R code facilitating the use of these packages is available in Huffaker et al. (2017). We used Origin 2022^39^ for 3-D plotting in Fig. 4. We modified R Code developed by Lukosevicius (2012) to run ESNN and wrote additional code employing high-performance computing and global sensitivity analysis to automate a large parallel grid search of ESNN architectural hyperparameters.
